# Salience of infectious diseases did not increase xenophobia during the COVID-19 pandemic

**DOI:** 10.1017/ehs.2024.28

**Published:** 2024-10-18

**Authors:** Lei Fan, Joshua M. Tybur, Paul A.M. Van Lange

**Affiliations:** a Vrije Universiteit Amsterdam, Department of Experimental and Applied Psychology, Van der Boechorststraat 7, 1081 BT, Amsterdam, the Netherlands; b CEPDISC - Centre for the Experimental-Philosophical study of Discrimination, Department of Political Science, Aarhus BSS, Aarhus University, Aarhus, Denmark; c Institute for Brain and Behavior Amsterdam Amsterdam, the Netherlands

**Keywords:** COVID-19, xenophobia, disgust, behavioral immune system, health

## Abstract

Multiple proposals suggest that xenophobia increases when infectious disease threats are salient. The current longitudinal study tested this hypothesis by examining whether and how anti-immigrant sentiments varied in the Netherlands across four time points during the COVID-19 pandemic (May 2020, February 2021, October 2021, and June 2022 through Flycatcher.eu). Results revealed that (1) anti-immigrant sentiments were no higher in early assessments, when COVID-19 hospitalizations and deaths were high, than in later assessments, when COVID-19 hospitalizations were low, and (2) within-person changes in explicit disease concerns and disgust sensitivity did not relate to anti-immigrant sentiments, though stable individual differences in disgust sensitivity did. These findings suggest that anecdotal accounts of increased xenophobia during the pandemic did not generalize to the population sampled from here. They also suggest that not all increases in ecological pathogen threats and disease salience increase xenophobia.

## 1 Introduction

Government policies during the COVID-19 pandemic drastically reduced the intermingling of individuals from different nations (e.g., Piccoli et al., 2021). Anecdotal accounts suggest that such policies co-occurred with broad increases in prejudice toward members of ethnic groups associated with foreign nationalities (e.g., Bhandari et al., 2021; Gover et al., 2020). Such anecdotes seemingly support hypotheses that humans respond to infectious disease outbreaks with greater xenophobia (e.g., Van Bavel et al., 2020; Schaller et al., 2022; Seitz et al., 2020). The current paper puts such hypotheses to the test by examining the degree to which attitudes toward immigration varied across the COVID-19 pandemic.

### 1.1 Intergroup contact and infectious disease

Many infectious disease outbreaks are imported from foreign lands. COVID-19 ravaged Chile after emerging in China; the Spanish Flu ravaged India after emerging in the U.S.; and the Black Death ravaged England after emerging in the Asian Steppe. The parasite stress literature proposes that these types of events have also occurred at smaller scales over the course of human evolution, with foraging groups on one side of a river or valley importing novel pathogens to neighboring groups (Thornhill & Fincher, 2014). A key proposition within this literature suggests that xenophobia has evolved in humans partially to reduce the infection threats posed by intergroup contact. Much of the empirical support for this proposition comes from findings indicating that values encouraging within-group (relative to between-group) assortment are higher in parts of the world with especially high parasite threats (see Fincher and Thornhill (2012), for an overview). Related studies have reported that implicit and explicit racial prejudice are higher in areas with higher infectious disease rates in the U.S. (O’Shea et al., 2019).

The behavioral immune system literature provides a complementary framework for understanding the proximate mechanisms that might lead individuals in higher parasite-stress ecologies to be more xenophobic (Fincher & Thornhill, 2012). It suggests that when disease-threats are salient, individuals feel more vulnerable to infection, and such perceptions of vulnerability feed into mechanisms that upregulate myriad anti-pathogen behaviors, including xenophobia and the tendency to feel disgust toward pathogen cues. Evidence for causal effects of disease salience on xenophobia comes from experimental studies reporting that negativity toward foreigners is higher among participants exposed to a disease-salience prime than among those exposed to a control prime (Faulkner et al., 2004; Huang et al., 2011; Klavina et al., 2011; Navarrete & Fessler, 2006). Complementary evidence is provided by studies using individual differences measures: those who report greater explicit disease concerns or who report more disgust toward pathogen cues endorse more negative attitudes toward foreigners (e.g., Aarøe et al., 2017; Brenner & Inbar, 2015; Clifford et al., 2022; Moran et al., 2021; Navarrete & Fessler, 2006).

Although designed to capture the same phenomenon, each of these methods has important limitations. Population-level studies have faced challenges in accounting for dependencies in units of analysis (Bromham et al., 2018; Currie & Mace, 2012), controlling for confounds (e.g., the fact that Western European institutions were established earlier in areas with low parasite stress; Hruschka & Henrich, 2013), and making inferences about individual-level psychology using group-level data (Pollet et al., 2014). This latter point is especially important, since it restricts the possibility of testing whether individuals who perceive more vulnerability to infection have more xenophobic attitudes. Although experiments priming disease salience via verbal stimuli (e.g., Huang et al., 2011) or visual stimuli (e.g., Faulkner et al., 2004; Klavina et al., 2011) are better equipped to make inferences about individual-level psychology, they also have limitations (Tybur et al., 2014). This validity of such procedures is not always clear, and recent well-powered pre-registered studies have not observed effects of such manipulations on intergroup biases (e.g., Makhanova et al., 2022) – an outcome that echoes the low replicability of social psychology studies using similar priming methods (e.g., Mac Giolla et al., 2022). Finally, all experimental studies in this area have used between-subjects designs (cf. Ko et al., 2024), which are unable to assess whether xenophobic attitudes vary within individuals as a function of disease salience.

### 1.2 Using the COVID-19 pandemic to test behavioral immune system hypotheses

The pandemic provided a unique opportunity for such a test concerning the effects of infectious disease on xenophobia. Hospitalizations and deaths from COVID-19 waxed and waned across time, and these changes reflected the likelihood of serious COVID-19 and, presumably, disease salience. These events allowed for research that overcomes limitations of existing cross-population and experimental priming studies. Specifically, they allowed for tests of whether average xenophobia within a population is higher when that population is experiencing a greater infectious disease threat, and they allowed for tests of whether individuals within that population report greater xenophobia when they feel more vulnerable to disease. Given the four-wave longitudinal nature of the current study, we were also able to address continuity and change in the primary variables (e.g., explicit disease concerns, pathogen disgust sensitivity, attitudes toward immigrants) across the pandemic.

In sum, this study provided a test of the hypothesis that attitudes toward immigrants become more negative as a function of pandemic severity. We further tested secondary hypotheses of why the relation between pathogen-avoidance psychology and anti-immigrant sentiments might be moderated by characteristics of the immigrant. One hypothesis suggests that pathogen-based xenophobia might be especially pronounced for immigrants who are more culturally dissimilar (e.g., Murray et al., 2011). Another hypothesis suggests that pathogen-based xenophobia might be especially pronounced for immigrants who offer fewer benefits as social partners (Tybur et al., 2020; Van Leeuwen et al., 2023). To test these secondary hypotheses, we described an immigrant that varied on his nationality, assimilation to the local culture, ability to provide benefits to locals, and willingness to provide benefits to locals before asking participants to report their attitudes toward immigrants like that target.

## 2 Method

### 2.1 Participants

Participants were recruited from the Netherlands via the survey agency Flycatcher (https://www.flycatcher.eu/). Data were collected at four time points across the COVID-19 pandemic at approximately eight-month intervals – May 2020, February 2021, October 2021, and June 2022. We excluded six participants who reported inconsistent demographic characteristics (sex, age, education level) across waves. The final sample included 1011 valid responses at Wave 1 (512 female), 724 at Wave 2 (352 female), 558 at Wave 3 (260 female), and 537 at Wave 4 (255 female). We determined the sample size based on our resources rather than a power analysis. Only participants who completed previous waves were invited to participate in Waves 2 and 3. All participants who completed (at least) Wave 1 were invited to complete Wave 4.

The study’s number of assessments was not pre-specified due to the unpredictability of the pandemic. Consequently, an a priori power analysis was not employed, nor was a pre-registration. We conclude data collection after Wave 4, after most of the population had acquired partial immunity due to vaccination and or infection, and after the most stringent government rules for reducing transmission had expired. A subsequent sensitivity analysis suggested that the design provided 80% power to detect differences in immigration attitudes and pathogen disgust sensitivity of approximately *d* = 0.08 and *d* = 0.10, respectively between Wave 1 and Wave 4.

### 2.2 Procedures and Materials

Participants followed the same procedure at each assessment. After providing consent, they were asked to complete a series of instruments, which took on average approximately 20 minutes. Those relevant to this paper concerned attitudes toward immigrants similar to the one described in a vignette (see below), explicit disease concerns, pathogen disgust sensitivity, and demographic variables (sex, age, and education level). The survey was conducted in Dutch. Materials were originally drafted in English and then they were translated from English to Dutch by a bilingual native Dutch speaker, back-translated into English by a bilingual native Dutch speakers, and checked by a native English speaker for consistency with the original items.

#### 2.2.1 Immigrant description

Participants first read about an immigrant, who was described as coming from one of 25 different nations and as having a name typical of his origin nation. For example, the immigrant from the United States was named James, the immigrant from China was named Yong, and the immigrant from South Korea was named Seojun. These nations were selected based on their cultural distances from the Netherlands (data were retrieved from Muthukrishna et al., 2020). We selected 20 nations at approximately equidistant cultural distance points from the Netherlands, and another five nations that allowed for better representation of world regions. The description of the immigrant included three other manipulations: (1) his ability to confer benefits to others (either coming from a wealthy family or from a poor family); (2) his motivation to confer benefits to others (either motivated improve their neighborhood or to amass possessions for himself); and (3) his assimilation to local norms (either rejecting Dutch social norms and maintaining those of their nation or origin or embracing Dutch social norms while rejecting those from their nation of origin). See online supplements for the details.

For each wave, nation of origin was sampled from the pool of 25 nations without replacement, and values each for the other three manipulations were randomly assigned.

#### 2.2.2 Immigration attitudes

After reading about the immigrant, participants first completed three manipulation check items assessing the immigrant’s economic status (“How wealthy is [TARGET] compared to the average person who grew up in the Netherlands?”), prosociality (“How interested in helping others is [TARGET] compared to the average person who grew up in the Netherlands?”), and norm assimilation (“To what degree has [TARGET] adopted Dutch norms, values, and traditions?”) on 7-point scales (for wealth, 1= much less wealthy to 7= much wealthier; for prosociality, 1 = not at all interested (in helping others) to 7 = very interested (in helping others); for norm assimilation, 1= not at all (adopting Dutch norms) to 7 = very much (adopting Dutch norms)). We also asked participants to rate the similarity between the immigrant’s origin nation and the Netherlands in terms of general culture, religious practices, cuisine, attitudes toward sex and romance, and health and hygiene on 7-point scales (1 = very dissimilar to 7 = very similar).

Following the manipulation checks, participants completed five items assessing their attitudes toward immigrants similar to the one described in the vignette on 7-point scales (e.g., “I feel positively toward people like [TARGET] immigrating to the Netherlands” and “I would support policies that allow people like [TARGET] to immigrate to the Netherlands”; 1-strongly disagree to 7 = strongly agree, Cronbach’s α = 0.93).

#### 2.2.2 Explicit disease concerns and pathogen disgust sensitivity

Explicit disease concerns were assessed using six items of the disease avoidance subscale from the Fundamental Motives Inventory (Neel et al., 2016), which were slightly modified to encourage participants to focus on current conditions (e.g., “I currently avoid places and people that might carry diseases” and “I am not very worried about getting germs from others right now”; 1= strongly disagree to 7= strongly agree, Cronbach’s α = 0.83). Participants then completed the Three-Domain Disgust Scale (TDDS, Tybur et al., 2009) in which they reported the degree to which they find each of 21 statements disgusting (1= not at all disgusting to 7= extremely disgusting). For the current study, we examined the seven-item pathogen subscale of the TDDS, which assesses disgust toward infection risks (e.g., “Shaking hands with a stranger who has sweaty palms” and “Stepping on dog poop” (Cronbach’s α = 0.77).

### 2.3 Analysis

Using linear-mixed models with maximal random-effects structures, we first tested whether explicit disease concerns, pathogen disgust sensitivity, and immigration attitudes varied across the pandemic. We regressed each of these variables on data collection wave while also modeling a random intercept for participants. For immigration attitudes, we also modeled a random intercept for origin nation. As a robustness check, we also applied Bayesian approach for re-testing our core results. Conclusions were similar. More details are provided in the SOM.

We also tested the effects of explicit disease concerns, pathogen disgust sensitivity, the three target manipulations (wealth, prosociality, and norm assimilation), and the cultural distance of the immigrant’s origin nation on immigration attitudes. We initially fitted the models with main effects of these variables, wave, and participant sex. In further analysis, we added interactions between data collection wave, explicit disease concerns, disgust sensitivity, and characteristics of the immigrant (see detailed description in each section).

## 3 Results

### 3.1 Manipulation checks

Participants perceived countries that are more culturally distant from the Netherlands as less similar to the Netherlands in terms of general culture (*β* = –0.30, 95% CI [–0.46, –0.14], *p* < 0.001), religious practices (*β* = –0.36, 95% CI [–0.49, –0.23], *p* < 0.001), cuisine (*β* = –0.25, 95% CI [–0.40, –0.10], *p* = 0.001), attitudes toward sex and romance (*β* = –0.33, 95% CI [–0.44, –0.21], *p* < 0.001), and health and hygiene (*β* = –0.20, 95% CI [–0.32, –0.08], *p* = 0.001). Target manipulations also worked as anticipated (for wealth, *M*_High_ = 4.75, 95%CI [4.64,4.85], *M*_Low_ = 2.56, 95%CI [2.45, 2.66], *t*(2772) = 44.71, *p* < 0.01, Cohen’s *d* = 1.70; for prosociality, *M*_Prosocial_ = 5.45, 95%CI [5.37, 5.52], *M*_Proself_ = 3.91, 95%CI [3.84, 3.98], *t*(2730) = 33.82, *p* < 0.01, Cohen’s *d* = 1.29; for norm assimilation, *M*_Adop_ = 5.21, 95%CI [5.13, 5.28], *M*_Reject_ = 2.64, 95%CI [2.56, 2.72], *t*(2707) = 51.20, *p* < 0.01, Cohen’s *d* = 1.97).

### 3.2 Explicit disease concerns and disgust sensitivity across waves

**Figure. 1:**
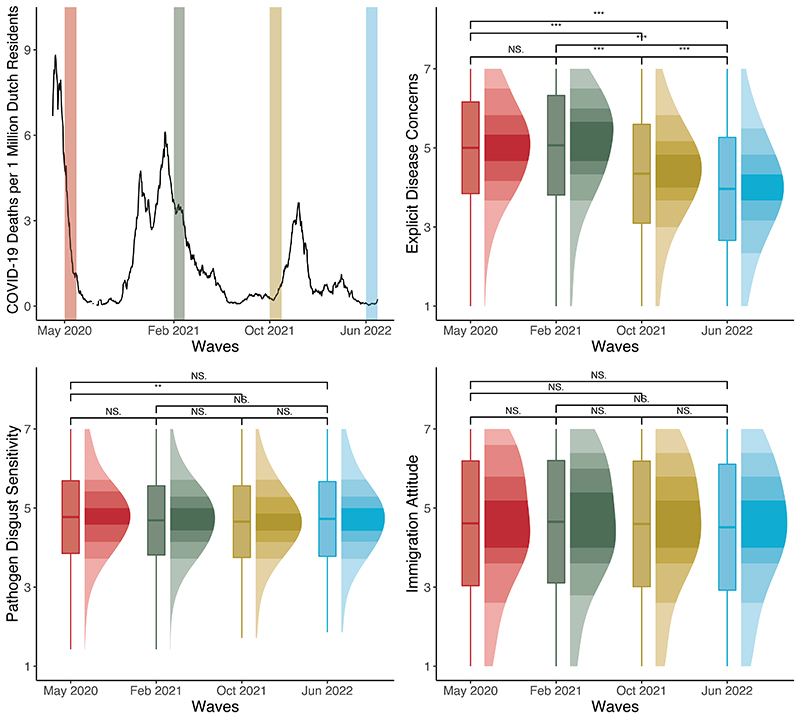
Official COVID-19 deaths, explicit disease concerns, pathogen disgust sensitivity, and immigration attitudes across timepoints during the pandemic. *Note*: * p < 0.05, ** *p* < 0.01***, *p* < 0.001. In each plot except the upper left, the horizontal line indicates the mean, the box indicates plus and minor one standard deviation, the whisker indicates the range, and the shaded area indicates the density of the data with different transparencies indicating quartiles. In the upper-left panel, the y-axis of COVID-19 deaths in the Netherlands refers to the daily new confirmed COVID-19 deaths per million people in the Netherlands. Data were retrieved from JHU CSSE COVID-19 Data (Dong et al., 2020). Color bars indicate the approximate survey window of each wave in the current study.

As expected, explicit disease concerns varied across waves, *F*(3, 2008) = 238.33, *p* < 0.001, η_*p*_^2^ = 0.26. They were much higher early in the pandemic, when COVID-19 mortality and hospitalizations remained high (*M*_W1_ = 5.00, 95% CI [4.93, 5.08]); *M*_W2_ = 5.03, 95% CI [4.95, 5.12]), than later in the pandemic, when immunity derived from vaccines and previous infection blunted hospitalizations and deaths (*M*_W3_ = 4.30, 95% CI [4.21, 4.40], *M*_W4_ = 3.91, 95% CI [3.82, 4.01]. In contrast, pathogen disgust sensitivity varied little across waves (*M*_W1_ = 4.77, 95% CI [4.72, 4.83], *M*_W2_ = 4.72, 95% CI [4.65, 4.78], *M*_W3_ = 4.68, 95% CI [4.62, 4.75], *M*_W4_ = 4.72, 95% CI [4.66, 4.79]), *F*(3, 1927) = 4.00, *p* = 0.01, η_*p*_^2^ = 0.01; see Figure 1).

### 3.3 Attitudes toward immigrants within and between waves

We did not detect differences in attitudes toward immigrants across the pandemic (*F*(3, 2066) = 0.99, *p* = 0.39, η*_p_*^2^ < 0.01, see Figure 1). Hence, although people felt more vulnerable to disease early in the pandemic relative to late in the pandemic, attitudes toward immigration remained similar across waves.

On average, attitudes toward immigrants were no different when disease concerns were, on average, relatively high (Waves 1 and 2) relative to when disease concerns were, on average, relatively low (Waves 3 and 4). However, this analysis does not address the association of within-person changes in pathogen-avoidance motivations with within-person changes in attitudes toward immigrants. To accomplish this task, we examined relations between attitudes toward immigrants and within-participant changes in explicit disease concerns and pathogen disgust sensitivity as well as person-level average of these two variables. We averaged values within participants across waves to represent stable disease concerns and pathogen disgust sensitivity, and we subtracted these within-participant means from each observation to estimate within-person change across waves. We then regressed immigration attitudes on both mean- and wave-varying scores for both explicit disease concerns and pathogen disgust sensitivity. Bivariate correlations between these and all other variables are shown in Table. 1.

We did not detect relations between attitudes toward immigrants and within-participant changes in either explicit disease concerns (*β* = 0.00, 95% CI [–0.03, 0.03], *p* = 0.91) or pathogen disgust sensitivity (*β* = –0.01, 95% CI [–0.03, 0.02], *p* = 0.53). Nor did we detect between-participant relations between explicit disease concerns and attitudes toward immigrants (*β* = 0.02, 95% CI [–0.03, 0.06], *p* = 0.49). We did, however, detect a relation between between-participant pathogen disgust sensitivity and immigration attitudes (*β* = –0.07, 95% CI [–0.11, –0.02], *p* < 0.01). These results suggest that changes in pathogen disgust sensitivity and explicit disease concerns across the pandemic did not relate to changes in immigration attitudes. However, stable individual differences in pathogen disgust sensitivity did relate to general negativity toward immigrants.

### 3.5 Moderating effects of immigrant characteristics

We detected effects of all three individual-level immigrant manipulations. Participants held more negative attitudes towards immigrants who come from a wealthy background (*M* = 4.56, 95% CI [4.48, 4.65]) than a poor background (*M* = 4.65, 95% CI [4.57, 4.74], *t*(2440) = 1.97, *p* = 0.05); those with pro-self orientations (*M* = 4.31, 95% CI [4.22, 4.39]) than pro-social orientations (*M* = 4.91, 95% CI [4.83, 5.00], *t*(2481) = 13.30, *p* < 0.001); and those who reject Dutch social norms (*M* = 3.92, 95% CI [3.84, 4.01]) rather than embrace them (*M* = 5.29, 95% CI [5.21, 5.38], *t*(2438) = 30.21, *p* < 0.001). We also observed a weak negative relation between cultural distance of the immigrant’s nation of origin and attitudes toward those immigrants (*β* = –0.03, 95% CI [–0.06, –0.00], *p* = 0.04).

**Figure. 2:**
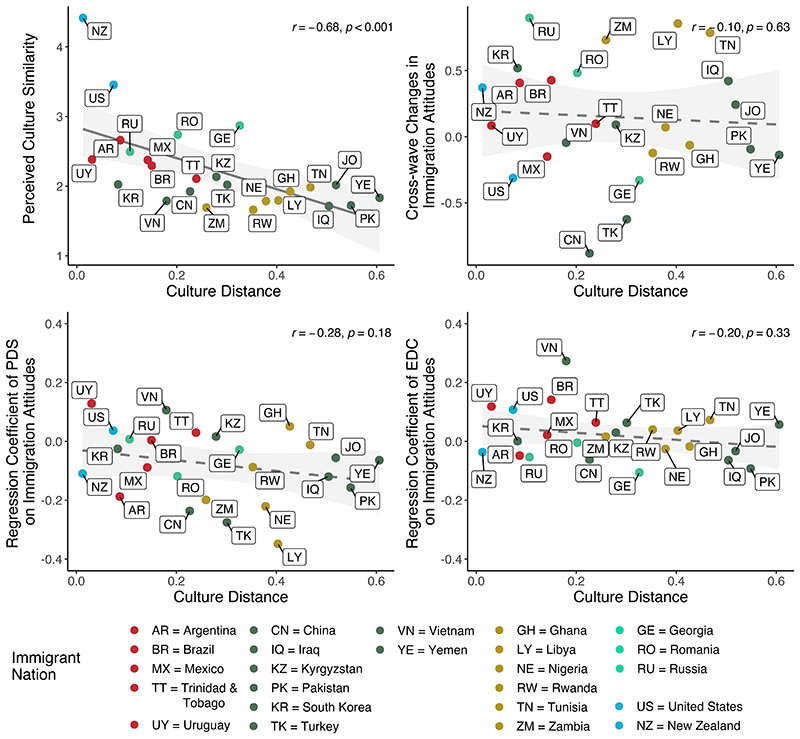
Relations between culture distance to the Netherlands, participants’ perceived culture similarity appraisal, cross-wave immigration attitudes changes, and regression coefficients between explicit disease concerns, pathogen disgust sensitivity and immigration attitude for each nation used in immigrant origin manipulation. *Note*: EDC = explicit disease concerns, PDS = pathogen disgust sensitivity. Cross-wave changes = (Wave 3 + Wave 4) – (Wave 1 + Wave 2). In each plot, the regression line indicates the zero-order regression of the variables on the axis. The shaded areas indicate their 95% CI. Correlations reported on top right refer to zero-order correlations between the variables in the plot. The coefficients plotted in the bottom two panels were post-hoc simple slope analyses results of the interaction terms of immigrant origin nation by EDC (*F*(24, 2250) = 0.78, *p* = 0.77, η_*p*_^2^ = 0.01) and PDS (*F*(24, 2283) = 0.90, *p* = 0.61, η_*p*_^2^ = 0.01). The models were with control of the fixed effect of all target attribute manipulations, waves, main effect of origin nation and the other individual difference variable, as well as the random effect of participants.

To test the secondary hypotheses described earlier, we further added interactions between target manipulations and explicit disease concerns, pathogen disgust sensitivity, and data collection wave. Only the interaction between person-average explicit disease concerns and the target norm assimilation manipulation was significant (*β* = –0.08, 95% CI [–0.13, –0.02], *t*(2393) = 2.62, *p* = 0.01); stable explicit disease concerns related to immigration attitudes when the immigrant was described complying with the local norms (*β* = 0.06, 95% CI [0.00, 0.11]), but not when rejecting them (*β* = -0.03, 95% CI [-0.09, 0.02]). We did not observe other significant interactions (see Fig. 2). Thus, we did not detect evidence that the features of the immigrants we manipulated moderated the relation between immigration attitudes and data collection time, explicit disease concerns, or disgust sensitivity.

## 4 Discussion

The current four-wave longitudinal study assessed whether xenophobia varied across the pandemic and whether it varied as a function of changes in explicit concerns about infectious disease and pathogen disgust sensitivity. Although explicit concerns about disease and deaths from COVID-19 were (much) higher earlier in the pandemic, negativity toward immigrants was not. Further, within-person changes in explicit disease concerns and disgust sensitivity did not correspond with changes in attitudes toward immigration. This finding appears incongruent with both anecdotal accounts of increased xenophobia during the pandemic and with findings in the behavioral immune system literature suggesting that disease salience and subjective vulnerability to infections increase xenophobia. We discuss implications of these findings for our understanding of how the pandemic affected individual psychology below.

### 4.1 COVID-19 pandemic and anti-immigration attitudes

Although media reports and anecdotal accounts suggest that prejudice toward ethnic groups associated with foreign nationalities increased during the pandemic, the current study did not detect such changes. The stability in anti-immigrant sentiments we observed mirrors results from some other recent multi-wave surveys. One study conducted in the U.S. detected an increase in Right-Wing Authoritarianism as COVID infections rose, but no increase in xenophobia (Pazhoohi & Kingstone, 2021). Another conducted in Germany detected no increase in stigmatization of Chinese and Asian-looking people (Koller et al., 2021). And another revealed that anti-immigrant prejudice in the Netherlands was no higher during in May 2020 than in 2017 (Muis & Reeskens, 2022). These findings echo conclusions from a systematical review indicating that the pandemic caused changes in feelings of threat but very limited changes in attitudes (Brandt et al., 2023). These (lack of) changes have especially important implications for how we understand the behavioral immune system.

### 4.2 The COVID-19 pandemic and the BIS

Disgust is often described as the motivational component of the behavioral immune system. Given predictions that the behavioral immune system should upregulate pathogen-avoidance motives when disease is salient, researchers have proposed that (pathogen) disgust sensitivity should increase during the pandemic. Some have interpreted data as supporting that proposal (e.g., Boggs et al., 2022; Stevenson et al., 2021), though others have reported small-to-no changes in disgust sensitivity (e.g., Carr et al., 2022; Miłkowska et al., 2021; Stefanczyk et al., 2024; Tybur et al., 2022). The current study was more in line with the second set of findings. And, although the current study did not include a pre-pandemic assessment, means in pathogen disgust sensitivity, which ranged from 4.68 to 4.77 across waves, were almost identical to (and, if anything, slightly lower than) the 4.82 reported in a similar sample recruited from the same survey company within the same country a decade earlier (Tybur & de Vries, 2013).

The lack of an effect of COVID-19 on pathogen disgust sensitivity has two broad theoretical implications. First, in contrast to a widespread treatment of disgust sensitivity and explicit disease concerns as interchangeable constructs (e.g., Oosterhoff et al., 2018), the two pathogen-avoidance variables seem differentially responsive to threats posed by COVID-19 (cf. Ackerman et al., 2021). Second, and related, the present findings suggest that the presence of a novel respiratory pathogen – as well as ubiquitous socially-transmitted information about that pathogen – probably does not act as input into whatever mechanisms output disgust responses.

Existing hypotheses have also suggested that the behavioral immune system might trigger or increase negativity toward only certain types of immigrants. We tested one such proposal by manipulating the origin nation of the immigrant and estimating the cultural distance between the origin nation and the host nation. Participants perceived more culturally distant nations as more culturally dissimilar. However, we did not detect interactions between cultural distance and time of assessment during the pandemic, explicit disease concerns, or disgust sensitivity. Similarly, pathogen-relevant variables largely did not moderate attitudes toward immigrants who were more versus less willing to provide resources, more versus less able to provide resources, and more versus less assimilated. This result raises questions regarding the robustness of previous findings indicating that pathogen-avoidance psychology only motivates antipathy toward certain types of immigrants (e.g., Faulkner et al., 2004; Karinen et al., 2019; Ji et al., 2019). In concert with findings that motivations to avoid interpersonal contact are much more strongly influenced by a target’s symptoms of infection and their relationship closeness rather than their ethnic or national group membership (Fan et al., 2022; Tybur et al., 2020; van Leeuwen & Peterson, 2018; also see Bressan, 2021, 2023), the current findings suggest that the social effects of the behavioral immune system might be more limited than previously proposed (see also Makhanova et al., 2022).

### 4.3 Implications for parasite stress theory

Parasite stress theory suggests that, over human evolutionary history, intergroup contact has posed greater infectious disease costs than intragroup contact, and that the relative risks of intergroup contact are greater in ecologies with greater parasite stress. Consequently, humans have evolved to respond to ecological parasite stress by adjusting attitudes, beliefs, and values in a way that increases intragroup assortment and decreases intergroup assortment (Fincher & Thornhill, 2012). This proposal has been debated. For example, some have pointed out that the relation between group membership and pathogen threat is not straightforward, since pathogens sometimes adapt to infect their local hosts (de Barra and Curtis, 2012). Further, even if outgroup pathogens are more dangerous, individual avoidance of outgroups might offer little protection against infection. If a single ingroup member interacts with outgroup members, the pathogen might spread to even those who avoided outgroups themselves. Hence, xenophobic individuals might miss out on all the benefits of intergroup interactions (e.g., trade, mating, knowledge transferring, access to territory) and still succumb to infections indirectly transmitted from outgroups (Fessler et al., 2015). Finally, parasite theory has been evaluated mostly by examining correlations between nation-level estimates of parasite stress and nation-level estimates of variables that putatively reflect preferences for intragroup assortment over intergroup assortment. This approach has been critiqued for multiple reasons, including its vulnerability to statistical artifacts (Bromham et al., 2018; Currie & Mace, 2012), its (in)ability to evaluate causal relationships (Hruschka & Henrich, 2013; Pollet et al., 2014), and its (in)ability to distinguish between evoked and transmitted cultural mechanisms (Schaller & Murray, 2012). The issue of evoked versus transmitted culture is especially relevant to the current study. Evoked culture would lead to cross-cultural variability because the features of the ecology taken as input by universal pathogen-avoidance adaptations vary across cultures; the latter would lead to the same variability because information and resulting norms are better transmitted, reproduced, and retained in some ecologies than others (Gangestad et al., 2006; Tooby & Cosmides, 1992).

The current study speaks against one possible route through which xenophobia could reflect evoked culture as implied by parasite stress theory. The introduction of virulent novel pathogen and ubiquitous social information about that pathogen did not lead to more negativity toward individuals from foreign ecologies. Hence, if variation in parasites indeed leads to variation in xenophobia, it does so via adaptations that take other information as input (e.g., inflammation; Fincher & Thornhill, 2012) or via transmitted culture. The latter could take at least two forms. First, norms that limit intergroup interactions could be better transmitted and retained if they give greater benefits in high parasite stress ecologies. Second, pathogen-avoidance psychology could act as a cultural attractor for the development of ethnocentric beliefs (Fessler & Machery, 2012), potentially as a byproduct of the lower social affordances of outgroup members relative to ingroup members (cf. Tybur et al., 2020). These beliefs could be advantageous for groups even if such advantages are unrelated to avoiding infection (Fessler et al., 2015). Naturally, more work is needed to test these ideas.

### 4.4 Limitations and future directions

We recruited our sample from the Netherlands – a nation that is relatively multicultural, liberal, and developed (Spiecker & Steutel, 2001). Inferences based on the current study might be limited to the population surveyed (or similar populations). As one example, attitudes toward poor immigrants were more positive than attitudes toward wealthy immigrants – a pattern that might emerge only in populations with economic and sociopolitical characteristics similar to that sampled from here. We did not have pre-pandemic data on this panel. Thus, while we can assess change from the early in the pandemic to a period after a widespread vaccination campaign, we cannot compare post-pandemic data to pre-pandemic data. The current study was not pre-registered, and we encourage secondary data analysis to verify the robustness of the conclusions reported here. Finally, the lack of an overall change in xenophobia does not preclude the possibility that the pandemic played a causal role in increasing intergroup violence perpetrated by a small number of individuals, even if attitudes toward immigrants did not change on average in the population sampled from. Naturally, the current findings do not speak to the impact or importance of the violence and harassment experienced by those targeted by such behaviors, regardless of such a causal relation.

While not suggesting that infectious disease is irrelevant to anti-immigrant sentiments, the current findings constrain the likely manner in which pathogens influence such prejudices. The presence of a novel pathogen (here, SARS-CoV-2), ubiquitous media attention to infectious disease, and shifts in subjective disease concerns do not appear to be the relevant inputs into any specialized psychological mechanisms that would output xenophobia – at least not in the population sampled from here. Any systematic shifts in prejudice instead might have been deployed toward ecological ingroup members who flaunted anti-COVID rules and recommendations (e.g., Republicans in the U.S.; Ko et al., 2024).

Ultimately, the scientific and societal merit of the present findings derives from contributing novel insights into understanding how pathogen threat relates to xenophobia. While the belief in an association seems widely shared among scientists the lay public, the present findings suggest that the association is not ubiquitous and certainly should not be considered a law of psychology. Future work in the parasite stress and behavioral immune system literatures can use these findings to triangulate upon the pathogen-relevant factors that might still contribute to xenophobia, even if a respiratory pandemic does not appear to be one of those factors. Regardless of the outcome of this research program, the current results offer some reassurance that the inevitable respiratory pandemics of the future need not lead cause widespread increases in xenophobia.

## Supplementary Material

Supplementary Materials

## Figures and Tables

**Table. 1:** Bivariate correlations (*N* = 2827)

Variables	1	2	3	4	5	6	7	8	9	10	11	12
1 Immigration attitudes	.93											
2 Explicit disease concerns	–.01	.83										
3 Pathogen disgust sensitivity	**–*.07***	***.19***	.77									
4 Survey wave ^a^	–.02	**–*.31***	–.03									
5 Wealth manipulation ^b^	**.05**	.02	–.02	.01								
6 Prosociality manipulation ^c^	**–*.18***	.03	.02	–.03	–							
7 Norm assimilation manipulation ^d^	**–*.43***	.02	–.00	.01	–	–						
8 EDC: between-participant	–.01	***.78***	***.23***	–.03	.03	.01	.02					
9 PDS: between-participant	**–*.08***	***.20***	***.90***	–.01	–.01	.01	–.01	***.25***				
10 EDC: within-participant	.00	***.63***	.02	**–*.45***	–.01	.03	.01	.00	.00			
11 PDS: within-participant	–.01	.03	***.43***	–*.05*	–.01	.01	.00	–.00	–.00	*.05*		
12 Cultural distance ^e^	–.02	–*.04*	.03	–.02	.00	.00	.00	–*.05*	.03	–.00	.01	
13 Participant sex ^f^	*.04*	***.08***	**.06**	–.03	–.01	.01	.01	***.10***	***.07***	.00	–.00	.00

*Note*: ***Bold and italics*** = *p* < 0.001, **bold** = *p* < 0.01, *italics* = *p* < 0.05, EDC = explicit disease concerns, PDS = pathogen disgust sensitivity. Cronbach’s alphas of multi-item measurements are on the diagonal.

a Spearman rather than Pearson correlations are reported for survey wave given its nature.

b For wealth manipulation, 0 = from a wealthy family, 1 = from a poor family.

c For prosociality manipulation, 0 = to improve their neighborhood, 1 = to amass possessions for himself.

d For assimilation to local norm manipulation, 0 = assimilating Dutch social norms, 1 = rejecting Dutch social norms.

e Culture distance refers to the distance between the origin nation of the immigrant target and the Netherlands, retrieved from Muthukrishna et al. (2020).

f For participant sex, 0 = male, 1 = female.

## Data Availability

Materials, data, and analysis scripts are available on OSF (https://osf.io/ghbaf/).

## Social Media summary

No rise in anti-immigrant sentiments in the Netherlands along COVID-19, no support for the disease-xenophobia link.
