# Supplementary material for: Salience of infectious diseases did not increase xenophobia during the COVID-19 pandemic
Source: Evol Hum Sci. Author manuscript; Available in PMC 2024 Dec 23. (PMC11514650; doi:10.1017/ehs.2024.28)
Supplement: Supplementary Materials [file EMS198166-supplement-Supplementary_Materials.pdf]

Supporting Online Material

This document provides additional information for the current paper. Table S1 lists the demographic composition of the sample, Table S2 displays the regression results of mixed-effect models testing the primary hypothesis on the relationship between pandemic severity, (within/between-participant) pathogen disgust sensitivity, and explicit disease concerns with attitudes towards immigrants, and Table S3 presents the results of regression models with further interactions to test the moderation effect from immigrant attributes and cultural distance to the focused prediction in the primary hypothesis. We also provided details of the sensitivity analysis, as well as the Bayesian regression results testing our main model shown with Table S4.

This document also includes information on the manipulations and measurements used in the project, including the immigration attitudes measurement task (Table S5 provides data on cultural distance) and the items for pathogen disgust sensitivity and explicit disease concerns measurements.

Demographical description of the current sample

Table S1. Demographical description of the current sample

Variables	*N* = 1011
Sex	
- Male	499
- Female	512
Age	
- 15-19	21
- 20-24	77
- 25-29	93
- 30-34	76
- 35-39	78
- 40-44	73
- 45-49	99
- 50-54	86
- 55-59	88
- 60-64	77
- >=65	243
Education	
- Elementary school	279
- Middle school	452
- College or University	280
Residing province	
- Drenthe	31
- Flevoland	27
- Friesland	30
- Gelderland	112
- Groningen	39
- Limburg	74
- Noord-Brabant	156
- Noor-Holland	153
- Overijssel	67
- Utrecht	82
- Zeeland	32
- Zuid-Holland	208

Regression Results of Main Analyses

Table S2. Regression results including main effects

Predictors	Immigration Attitudes					
	β	95% CI	*p*	*β*	95% CI	*p*
(Intercept)	.56	.48, .65	<.001	.56	.48, .65	<.001
Wealth manipulation	.06	.00, .11	.05	.06	.00, .11	.05
Prosociality manipulation	–.39	–.44, –.33	<.001	–.39	–.44, –.33	<.001
Norm assimilation manipulation	–.87	–.92, –.81	<.001	–.87	–.92, –.81	<.001
EDC	.01	–.03, .04	.69			
PDS	–.05	–.09, –.01	.01			
Cultural distance	–.03	–.06, –.00	.04	–.03	–.06, –.00	.05
Survey wave [2]	.05	–.02, .12	.14	.05	–.02, .12	.14
Survey wave [3]	.00	–.07, .08	.94	.00	–.07, .08	.95
Survey wave [4]	–.02	–.10, .06	.58	–.03	–.11, .06	.55
Participant sex	.07	–.02, .15	.15	.07	–.02, .16	.14
Participant age	–.07	–.11, –.03	<.01	–.07	–.11, –.02	<.01
EDC: between-participant				.02	–.03, .06	.51
PDS: between-participant				–.07	–.11, –.02	<.01
EDC: within-participant				.00	–.03, .03	.91
PDS: within-participant				–.01	–.03, .02	.53
**Random Effects**						
σ^2	1.12			1.12		
$\tau_{00\ ID}$	0.77			0.76		
$\tau_{00\ Origin\ Nation}$				0.00		
ICC	0.41			0.41		
$N_{Origin\ Nation}$				25		
N_{ID}	1011			1011		
Observations	2827			2827		
Marginal / Conditional R^2	0.236 / 0.547			0.238 / 0.548		

Note: EDC = explicit disease concerns, PDS = pathogen disgust sensitivity.

Table S3. Regression results of secondary model including interactions of EDC, PVD and survey wave with immigrant target attribute manipulations and cultural distance

	Immigration Attitudes		
Predictors	*β*	95% CI	*p*
(Intercept)	.58	.47, .69	<.001
Wealth manipulation	.08	–.02, .17	.12
Prosociality manipulation	–.37	–.47, –.27	<.001
Norm assimilation manipulation	–.95	–1.04, –.85	<.001
Cultural distance	.00	–.04, .05	.87
EDC	.03	–.03, .10	.34
PDS	–.02	–.08, .04	.52
Survey wave [2]	.03	–.12, .17	.74
Survey wave [3]	–.03	–.19, .13	.74
Survey wave [4]	–.05	–.22, .12	.57
Participant sex	.07	–.02, .16	.14
Participant age	–.07	–.12, –.03	.002
Wealth manipulation * EDC	.00	–.06, .06	.94
Wealth manipulation * PDS	–.03	–.09, .02	.26
Wealth manipulation * Survey wave [2]	–.02	–.17, .13	.81
Wealth manipulation * Survey wave [3]	–.01	–.17, .15	.88
Wealth manipulation * Survey wave [4]	–.06	–.23, .11	.50
Prosociality manipulation * EDC	.02	–.04, .08	.56
Prosociality manipulation * PDS	–.04	–.09, .02	.21
Prosociality manipulation * Survey wave [2]	–.02	–.17, .12	.77
Prosociality manipulation * Survey wave [3]	–.07	–.23, .09	.42
Prosociality manipulation * Survey wave [4]	.03	–.13, .20	.69
Norm assimilation manipulation * EDC	–.07	–.13, –.01	.03
Norm assimilation manipulation * PDS	.01	–.05, .06	.84
Norm assimilation manipulation * Survey wave [2]	.09	–.05, .24	.21
Norm assimilation manipulation * Survey wave [3]	.16	–.00, .32	.06
Norm assimilation manipulation * Survey wave [4]	.09	–.08, .26	.29
Cultural distance * EDC	–.01	–.05, .02	.36
Cultural distance * PDS	–.02	–.05, .01	.14
Cultural distance * Survey wave [2]	–.05	–.13, .02	.17
Cultural distance * Survey wave [3]	–.08	–.16, –.00	.05
Cultural distance * Survey wave [4]	–.02	–.10, .06	.64
**Random Effects**			
σ^2	1.12		
$\tau_{00\ ID}$	0.77		
ICC	0.41		
N_{ID}	1011		
Observations	2827		
Marginal / Conditional R^2	0.240 / 0.551		

Note: EDC = explicit disease concerns, PDS = pathogen disgust sensitivity.

Sensitivity Analysis

To estimate statistical power, we executed a sensitivity analysis using the 'simr' package. We initiated this process by retrieving data collected from Waves 1 and 4. Using these data, we regressed the variables of interest - pathogen disgust sensitivity and immigration attitudes - on the fixed effect of Wave, and we used the random intercept of participant ID. We ran 1000 simulations for each test, with a significance level of 0.05, and we iteratively changed the effect size until we arrived at a power of 80%. Results indicated that we had 80% power to detect a small effect size for the cross-wave variations of pathogen disgust sensitivity (r_{partial} = 0.04, Cohen's d = 0.08) and immigration attitudes (r_{partial} = 0.05, Cohen’s d = 0.10) between Wave 1 and Wave 4.

Bayesian Modeling

We fitted a Bayesian linear mixed model (estimated using MCMC sampling with 4 chains of 2000 iterations and a warmup of 1000) to predict immigration attitudes with three immigrant manipulations, explicit disease concerns, pathogen disgust sensitivity, cultural distance, wave, and participant sex and age. The model included participant ID as a random intercept. Parameter priors were set as normal distributions. The model's explanatory power was substantial (R^2 = 0.55, 95% CI [0.53, 0.58], adjusted R^2 = 0.42) and the part related to the fixed effects alone (marginal R^2) is of 0.24 (95% CI [0.21, 0.26]).

The effect of wealth manipulation has a 97.65% probability of being positive (> 0), 59.62% of being significant (> 0.08), and 0.00% of being large (> 0.47). The estimation successfully converged (Rhat = 1.000) and the indices are reliable (ESS = 3280); the effect of prosociality manipulation has a 100.00% probability of being negative (< 0), 100.00% of being significant (< -0.08), and 99.80% of being large (< -0.47). The estimation successfully converged (Rhat = 1.000) and the indices are reliable (ESS = 3106); the effect of norm assimilation manipulation has a 100.00% probability of being negative (< 0), 100.00% of being significant (< -0.08), and 100.00% of being large (< -0.47). The estimation successfully converged (Rhat = 0.999) and the indices are reliable (ESS = 3137)

The effect of explicit disease concerns has a 65.18% probability of being positive (> 0), 0.10% of being significant (> 0.08), and 0.00% of being large (> 0.47). The estimation successfully converged (Rhat = 1.005) and the indices are reliable (ESS =

1526). The effect of pathogen disgust sensitivity has a 99.65% probability of being negative (< 0), 62.42% of being significant (< -0.08), and 0.00% of being large (< -0.47). The estimation successfully converged (Rhat = 1.002) and the indices are reliable (ESS = 1039)

The effect of cultural distance has a 97.75% probability of being negative (< 0), 91.95% of being significant (< -0.08), and 5.67% of being large (< -0.47). The estimation successfully converged (Rhat = 1.000) and the indices are reliable (ESS = 1139)

Compare with Wave 1, the effect of Wave 2 has a 92.27% probability of being positive (> 0), 50.52% of being significant (> 0.08), and 0.00% of being large (> 0.47). The estimation successfully converged (Rhat = 1.000) and the indices are reliable (ESS = 3762); Wave 3 has a 53.73% probability of being positive (> 0), 11.55% of being significant (> 0.08), and 0.00% of being large (> 0.47). The estimation successfully converged (Rhat = 1.004) and the indices are reliable (ESS = 1446); Wave 4 has a 69.08% probability of being negative (< 0), 25.37% of being significant (< -0.08), and 0.00% of being large (< -0.47). The estimation successfully converged (Rhat = 1.000) and the indices are reliable (ESS = 2959)

The effect of participant sex has a 91.92% probability of being positive (> 0), 61.55% of being significant (> 0.08), and 0.00% of being large (> 0.47). The estimation successfully converged (Rhat = 1.001) and the indices are reliable (ESS = 2249); participant age has a 99.88% probability of being negative (< 0), 0.00% of being

significant (< -0.08), and 0.00% of being large (< -0.47). The estimation successfully converged (Rhat = 1.002) and the indices are reliable (ESS = 1988)

Table S4. Bayesian regression results of the main model

	Immigration Attitudes	
Predictors	*B*	95% CI
(Intercept)	6.25	5.84, 6.66
Wealth manipulation	0.09	0.00, 0.18
Prosociality manipulation	–0.61	–0.70, –0.52
Norm assimilation manipulation	–1.37	–1.46, –1.28
Cultural distance	–0.27	–0.53, –0.00
Explicit disease concern	0.01	–0.03, 0.05
Pathogen disgust sensitivity	–0.09	–0.16, –0.02
Survey wave [2]	0.08	–0.03, 0.18
Survey wave [3]	0.00	–0.11, 0.12
Survey wave [4]	–0.04	–0.17, 0.09
Participant sex	0.10	–0.04, 0.24
Participant age	–0.04	–0.06, –0.01
**Random Effects**		
σ^2	1.12	
$\tau_{00\ ID}$	0.77	
ICC	0.41	
N_{ID}	1011	
Observations	2827	
Marginal / Conditional R^2	0.237 / 0.551	

Immigration attitudes measurement task

Immigrant instructions

Great! You are done with the first part of the survey. For the next part, you will read a description of a person who has immigrated to the Netherlands, and you will answer some questions about your opinions about immigrants like this person. XXX is a man from ZZZ who has recently immigrated to the Netherlands. XXX (comes from a wealthy family and has moved to the Netherlands with more than enough money to get by)/(comes from a poor family and has moved to the Netherlands with almost no money). Now that he is in the Netherlands, he is strongly motivated to (help others and make his neighborhood a better place)/(accumulate as much money and possessions as possible for himself). XXX does everything he can to live by the norms, values, and traditions (of the Dutch/that he was raised with in ZZZ), even if doing so means rejecting the norms, values, and traditions that are common in (the Netherlands/ZZZ).

Wealth manipulation

– comes from a wealthy family and has moved to the Netherlands with more than enough money to get by

– comes from a poor family and has moved to the Netherlands with almost no money

Prosociality manipulation

– comes from a wealthy family and has moved to the Netherlands with more than enough money to get by

– comes from a poor family and has moved to the Netherlands with almost no money

Norm assimilation manipulation

– does everything he can to live by the norms, values, and traditions of the Dutch, even if doing so means rejecting the norms, values, and traditions that are common in (IMNATION).

– does everything he can to live by the norms, values, and traditions of (IMNATION), even if doing so means rejecting the norms, values, and traditions that are common in the Netherlands.

Origin Nation manipulation

Table S4. Immigrant origin nation manipulation and cultural distance

Nation	Name	Cultural Distance from the Netherlands
New Zealand	Oliver	0.012
Uruguay	Augastin	0.03
United States	James	0.074
South Korea	Seo-jun	0.083
Argentina	Pedro	0.087
Russia	Artyom	0.106
Mexico	Mateo	0.142
Brazil	Marcos	0.15
Vietnam	Tuan	0.18
Romania	Alexandru	0.202
China	Yong	0.227
Trinidad and Tobago	Ceejay	0.239
Zambia	Mulubwa	0.259
Kyrgyzstan	Vladislav	0.279
Turkey	Hasan	0.301
Georgia	Nikoloz	0.326
Rwanda	Dido	0.353
Nigeria	Osita	0.378
Libya	Mahmoud	0.403
Ghana	Emmanuel	0.427
Tunisia	Mehdi	0.468
Iraq	Mahdi	0.504
Jordan	Ahmad	0.519
Pakistan	Zahid	0.549
Yemen	Fahd	0.606

Immigration Attitudes Items

1. How wealthy is XXX compared to the average person who grew up in the Netherlands?

2. How interested in helping others is XXX compared to the average person who grew up in the Netherlands?

3. To what degree has XXX adopted Dutch norms, values, and traditions?

4. I feel positively toward people like XXX immigrating to the Netherlands.

5. XXX is the type of immigrant I would welcome in the Netherlands.

6. I would support policies that allow people like XXX to immigrate to the Netherlands.

7. I oppose people like XXX immigrating to the Netherlands.

8. People like XXX make our country worse.

9. XXX is from ZZZ. In general, how similar are ZZZ and the Netherlands in terms of culture?

10. XXX is from ZZZ. In general, how similar are ZZZ and the Netherlands in terms of religious practices?

11. XXX is from ZZZ. In general, how similar are ZZZ and the Netherlands in terms of cuisine?

12. XXX is from ZZZ. In general, how similar are ZZZ and the Netherlands in terms of attitudes toward sex and romance?

13. XXX is from ZZZ. How similar are ZZZ and the Netherlands in terms of health and hygiene?

Items 1-3 are manipulation checks for immigrant's attribute manipulation; Items 4-8 are the core measurement items of immigration attitudes which was used in the main analysis; Items 9-13 are manipulation checks for the cultural distance manipulation. All items were on Likert-like scales of 1-7.

Item 1: 1 - Much less wealthy to 7 – Much wealthier

Item 2: 1 - Not at all interest to 7 – Very interested

Item 3: 1 - Not at all – to 7 - Very much

Items 4-8: 1 - Strongly disagree to 7 – Strongly agree

Items 9-13: 1 – Very dissimilar to 7 – Very similar

Explicit concern measurement items

Great! You are done with the second part of the survey. The rest of the survey includes questions about your views toward society and relationships between groups, emotional reactions to situations, and issues related to infectious disease and the economy. In total, there are XX questions remaining, and they should take you about another six or seven minutes to complete.

Some of these questions ask you specifically about your current thoughts (rather than your thoughts in general). For these questions, please make sure to think about your current thoughts.

1. I currently avoid places and people that might carry diseases

2. I currently avoid people who might have a contagious illness

3. I am currently worried about catching a disease from too much contact with other people

4. I am not very worried about getting germs from others right now

5. If someone near me were sick right now, I wouldn't be very bothered

6. Right now, I wouldn't mind being around someone who is sick

All items were on Likert-like scales of 1 - Strongly disagree to 7 – Strongly agree.

Pathogen disgust sensitivity items

The following items describe a variety of concepts. Please rate how disgusting you find the concepts described in the items, where 1 means that you do not find the concept disgusting at all and 7 means that you find the concept extremely disgusting.

1. Stepping on dog poop
2. Sitting next to someone who has red sores on their arm
3. Shaking hands with a stranger who has sweaty palms
4. Seeing some mold on old leftovers in your refrigerator
5. Standing close to a person who has body odor
6. Seeing a cockroach run across the floor
7. Accidentally touching a person's bloody cut

All items were on Likert-like scales 1 – not at all disgusting to 7 – extremely disgusting
